# The Management of Ruptured Abdominal Aortic Aneurysms: An Ongoing Challenge

**DOI:** 10.3390/jcm12175530

**Published:** 2023-08-25

**Authors:** Nicola Troisi, Giulia Bertagna, Lorenzo Torri, Francesco Canovaro, Mario D’Oria, Daniele Adami, Raffaella Berchiolli

**Affiliations:** 1 Vascular Surgery Unit, Department of Translational Research and New Technologies in Medicine and Surgery, University of Pisa, 56126 Pisa, Italy; giuliaberty.it@hotmail.it (G.B.); lorenzo.torri@gmail.com (L.T.); canovarofrancesco94@gmail.com (F.C.); danieleadami71@gmail.com (D.A.); raffaella.berchiolli@unipi.it (R.B.); 2 Vascular Surgery Unit, Azienda Sanitaria Universitaria Giuliano Isontina, 34148 Trieste, Italy; mario.doria88@outlook.com

**Keywords:** ruptured abdominal aortic aneurysm, open surgical repair, endovascular repair, high-volume center, preoperative scores

## Abstract

Background: despite improvements in the diagnosis and treatment of elective AAAs, ruptured abdominal aortic aneurysms (RAAAs) continue to cause a substantial number of deaths. The choice between an open or endovascular approach remains a challenge, as does postoperative complications in survivors. The aim of this manuscript is to offer an overview of the contemporary management of RAAA patients, with a focus on preoperative and intraoperative factors that could help surgeons provide more appropriate treatment. Methods: we performed a search on MEDLINE, Embase, and Scopus from 1 January 1985 to 1 May 2023 and reviewed SVS and ESVS guidelines. A total of 278 articles were screened, but only those with data available on ruptured aneurysms’ incidence and prevalence, preoperative scores, and mortality rates after emergency endovascular or open repair for ruptured AAA were included in the narrative synthesis. Articles were not restricted due to the designs of the studies. Results: the centralization of RAAAs has improved outcomes after both surgical and endovascular repair. Preoperative mortality risk scores and knowledge of intraoperative factors influencing mortality could help surgeons with decision-making, although there is still no consensus about the best treatment. Complications continue to be an issue in patients surviving intervention. Conclusions: RAAA still represents a life-threatening condition, with high mortality rates. Effective screening and centralization matched with adequate preoperative risk–benefit assessment may improve outcomes.

## 1. Introduction

Abdominal aortic aneurysm (AAA) is a relatively frequent pathology with a global prevalence up to 8%. It is a major cause of death in the United States, in the United Kingdom, and in Europe, with a higher risk among individuals older than 60 years [1,2,3]. Previous estimates in developed countries have shown that AAA rupture causes from 53% to 65% of in-hospital and 43% of interventional deaths. Consequently, the Centers for Disease Control classified AAA as the 15th cause of death in the United States [4]. Despite improvements in early diagnosis and treatment of elective AAA, ruptured abdominal aortic aneurysms (RAAAs) continue to cause a substantial number of deaths. Furthermore, the introduction of endovascular aortic repair (EVAR) has contributed to increases in elective AAA repair. This results in more patients with asymptomatic AAA being offered elective repair before rupture occurs [5,6]. However, mortality resulting from RAAA is influenced by multiple factors, including the promptness of patients’ transfers to the hospital, eligibility for surgical repair, perioperative mortality, presence of well-defined RAAA pathways, and treatment performed in high-volume centers [7].

Indeed, approximately two-thirds of patients with RAAA die before reaching the hospital, and the remaining 20% die before transfer to the theatre [8,9]. Although intraoperative mortality has reduced due to improved anesthetic and intensive care management postoperatively in the past two decades, the overall 30-day mortality remains unchanged. This is probably related to the aging patient population and comorbidities, with approximately 50% dying in the intensive care unit (ICU) following postoperative complications, leading quite frequently to multi-organ failure (MOF) [10,11].

Treatment of RAAA includes open surgical repair (rOSR) and endovascular aneurysm repair (rEVAR). EVAR has now become a valid treatment option for elective AAA repair, particularly in high-risk patients and in those with a hostile abdomen, and has also shown encouraging outcomes in the emergency setting [12]. However, these findings are inadequate to support EVAR as the gold standard for RAAA patients [13]. Comparative studies between rOSR and rEVAR have been developed to assess the efficacy, complications, and cost-effectiveness of both techniques [14,15]. However, the majority of these studies were noncomprehensive or outdated, leading to heterogeneous results.

Age and sex are important risk factors for RAAA, influencing the treatment approach. Elderly patients previously deemed inoperable because of high surgical risk can now be considered for EVAR, which is demonstrated to have lower perioperative mortality [16]. Gender-related issues have been addressed by technological advancements, which have broadened EVAR indications in women. Nevertheless, RAAA is still a challenging condition with significant morbidity and mortality. Advances in surgical techniques, endovascular repair, and perioperative care have improved outcomes, but further research is necessary to optimize treatment strategies and reduce mortality rates. As for stroke, “time is brain” similarly, for RAAA, “time is blood”, assuming that better results are achieved with prompt diagnosis and treatment. Therefore, great attention is essential to identify signs and symptoms of RAAA before hemodynamic instability, which is known to worsen the prognosis. Reducing the time between symptoms and treatment, with protocols, well-trained triages, and efficient transports to high-volume centers, is mandatory to achieve satisfactory outcomes.

## 2. Materials and Methods

The present review was conducted and reported in accordance with the Preferred Reporting Items for Systematic Reviews and Meta-Analyses (PRISMA) guidelines. The literature search strategy was carried out by the review author team. Preliminary searches were conducted on Medical Literature Analysis and Retrieval System Online (MEDLINE), Excerpta Medica Database (EMBASE), and Scopus from 1 January 1985 to 1 May 2023. SVS and ESVS guidelines were reviewed as well. A combination of controlled vocabulary and free text terms was used to investigate the databases. Specifically, the following search strategies were used on each database:

Abdominal aortic aneurysm and rupture and (open surgical repair OR endovascular repair OR EVAR) and (preoperative and (assessment and (assessment OR score))) (textword).

Where possible, abstracts were reviewed online and suitable articles downloaded for data extraction. If abstracts were not available, a full copy of the article was assessed. The main inclusion criterion was the availability of data on ruptured aneurysms’ incidence and prevalence, preoperative scores and mortality rates after emergency EVAR or open repair for ruptured AAA. Articles were not restricted due to design of study (retrospective, prospective, observational, etc.). Furthermore, no language constraints were applied to the research.

Reasons for exclusion:

Reason 1: no 30-day mortality data about RAAA treated both open and endovascularly.

Reason 2: data about emergency and elective AAA repair were merged.

Reason 3: no endovascular repair offered.

**Figure 1:**
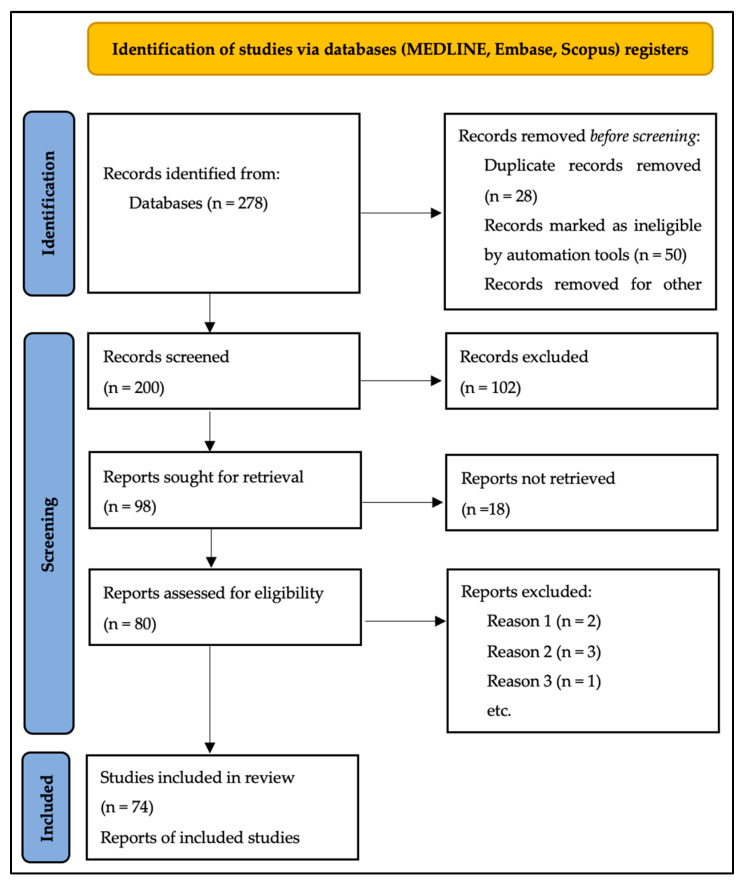
Study flow chart.

Information extracted are reported in Table 1 based on PICOS approach:

The study selection process is presented in a flow diagram following PRISMA criteria (Figure 1).

## 3. Results

The literature search, applying the defined aforementioned strategy, retrieved 278 reports. Of those, 78 studies fulfilled the inclusion criteria. Three studies reported data which do not fulfill the inclusion criteria; therefore, they were excluded from the present analyses.

All studies included underline the importance of centralization in the overall management of RAAA. Indeed, the centralization of RAAA patients in high-volume centers has improved outcomes after both surgical and endovascular repair. These promising results seem to be more pronounced for rOSR, where surgeons’ volume plays a pivotal role as well. Moreover, the development of preoperative mortality risk scores has improved patient selection and perioperative survival. However, the usefulness of all risk scores resides more in the information they can provide, which can help clinical decision-making before treatment, rather than in giving a measure of perioperative mortality, in order to deny surgical treatment. Furthermore, during the procedure, be it open or endovascular, knowledge of intraoperative factors influencing mortality could help surgeons with decision-making, although there is still no consensus about the best treatment. Obviously, among survivors, complications continue to be an issue. EVAR usually has lower rates of short-term complications than rOSR, but more mid-term reinterventions related to endoleaks. When OSR is performed, pulmonary complications are the most common, while bowel ischemia is the most threatening one due to its high mortality rates.

## 4. Discussion

The centralization of care, which implies transferring patients to referral centers with experienced teams and adequate resources, has been largely investigated. This section explores the importance of RAAA centralization and highlights its benefit in terms of outcomes, mortality rates, and healthcare resource utilization [17].

Centralization refers to the consolidation of specialized health services or procedures in selected centers. For RAAA, centralization involves directing patients to specific centers that own expertise, infrastructures, and resources for providing optimal care.

RAAAs require rapid assessment and immediate intervention to control bleeding. Then, repairing the aneurysm and preventing further complications requires the following compulsory steps. The entire procedure requires a multidisciplinary approach involving vascular surgeons, anesthesiologists, intensive care specialists, interventional radiologists, general surgeons, and other specialists. Moreover, the availability of appropriate equipment is essential for effective treatment. Centralization of care for RAAA has significantly improved patients’ outcomes [7,17]. Several studies have demonstrated that patients treated in referral centers had lower mortality and complication rates, and shorter hospital stays, compared to those treated in non-centralized settings [7,18]. A subgroup analysis showed lower perioperative mortality in high-volume centers for both EVAR and OSR, with a more pronounced effect in the open surgery subgroup. Furthermore, sensitivity analysis of contemporary studies did not show a significant survival advantage in high-volume centers for EVAR [8,18]. This suggests that a high annual RAAA volume treated with EVAR may not significantly affect outcomes. Surely, concentration of expertise and resources leads to more efficient and effective care delivery.

Healthcare professionals working in these centers have adequate knowledge and skills to manage complications associated with this condition. Surgeons performing a higher volume of interventions seem to have better outcomes [17]. However, evidence regarding the impact of an individual surgeon’s caseload on outcomes following surgery is less robust than that about institutional volume, and it is more evident for OSR. The individual surgeon is a small (albeit significant) part in the patient’s journey, and the overall healthcare infrastructure may have a greater impact on clinical outcomes.

Therefore, the concentration of aortic services could have a profound influence on patient care, outcomes, and their overall experience. Ensuring round-the-clock availability of aortic services implies various modifications in logistics, infrastructure, and healthcare delivery. This includes prehospital care, diagnostic imaging, surgical facilities (such as hybrid theatre), aortic endografts, supplementary equipment, postoperative intensive care management, and the presence of an experienced vascular multidisciplinary team. However, it could be argued that the transfer to a referral aortic center may prolong the process, potentially affecting overall mortality rates, since some patients may not receive timely surgical intervention.

Another important issue concerns streamlined pathways. Centralization promotes the development of standardized care pathways and protocols specific for RAAAs. This ensures appropriate diagnosis, treatment, and postoperative management, leading to improved quality of care. Eventually, resource utilization remains one of the key points for the healthcare system. Referral centers can invest in cutting-edge equipment, including advanced imaging technologies and endovascular devices. Concentrating resources in a limited number of centers ensures the optimal use of expensive equipment, reduces redundant investments, and promotes cost-effectiveness.

**Figure 2:**
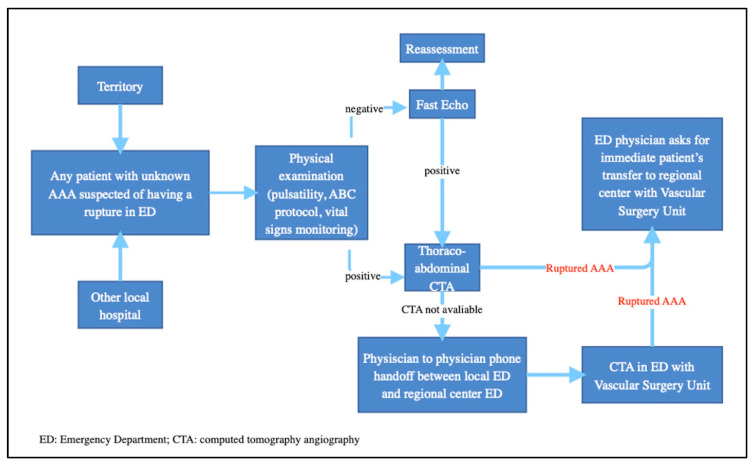
The flow chart summarizes the centralization process.

Centralization to high-volume hospitals, often defined as the “hub and spoke” model, can encounter several challenges. The first one is geographic accessibility, which constitutes an issue, especially for patients living in remote areas, so that establishing effective networks and transport to referral centers is crucial. Telemedicine and teleconsultation can also play a role in improving access to specialists, enabling remote assessment and guidance. Secondly, another main challenge concerns transport networks, including ambulances, helicopters, or other transport modalities, as well as communication between emergency services and central hospitals. On the other hand, centralization inevitably leads to a higher patient flow to the index hospital, which can strain its capacity and resource management. Therefore, careful planning, ensuring an efficient management of patient flow without compromising the quality of care, is mandatory (Figure 2).

Another issue concerns team training, so that significant investments are needed to adequately train team members and ensure that their skills remain up-to-date. In this context, cooperation among different centers is essential to ensure efficient patient flow and accurate communications between medical teams.

Despite possible improvements in all the abovementioned fields, patient acceptance and trust in physicians is mandatory. Centralization requires a shift in patients’ culture and habits, so that they may accept emergency care in the closest hospital. Patient trust in the new centralization model takes time to develop and can be influenced by perceptions of accessibility, quality of care, and safety.

Standardized protocols enable high-volume hospitals to establish streamlined processes for prompt intervention. These protocols often include guidelines for rapid triage, advanced imaging techniques, and access to hybrid theatres equipped with current technologies. Efficient diagnosis and timely intervention reduce treatment delay, resulting in improved patient outcomes and increased survival rates.

The centralization protocols have the following objectives:

1. Standardizing the approach: this ensures that all patients receive uniform care, based on best clinical practice, regardless of the hospital facility in which they are treated.
2. Reducing time for intervention: this involves the rapid diagnosis and efficient planning of either surgical or endovascular intervention. Timely intervention is crucial to improve short- and long-term outcomes and to increase the chances of a patient’s survival.
3. Improving clinical outcomes: this may include reducing intraoperative and postoperative mortality, decreasing complications, improving pain management, and quicker recovery. Centralization also enables the systematic collection of clinical data, which helps to monitor outcomes and further improve clinical practice.
4. Quality assurance and standardization: these protocols are developed on the basis of evidence-based practice, national guidelines, and clinical expertise. Implementation of standardized protocols through multiple hospitals enhances care delivery and reduces discrepancies in treatment approaches. Regular audits and quality control measures help to identify areas that require improvement and to refine protocols for further enhancing patient care.
5. Data collection and research opportunities: standardized protocols ease the collection of comprehensive clinical data, including patient demographic features, treatment outcomes, and long-term follow-up. These data serve as a valuable resource for research studies, evaluating the effectiveness of different interventions, and identifying trends or areas that require further investigation.
6. Training and education: high-volume centers offer training programs, fellowships, and educational opportunities for medical students, residents, and fellows. In the context of a learning environment, centralization promotes expertise and encourages the dissemination of knowledge throughout the medical community.

In conclusion, centralization protocols for RAAA treatment aim to standardize care, improve access to specialized centers, reduce intervention times, enhance clinical outcomes, and optimize resource usage in order to provide the best treatment for each individual patient.

Despite indisputable advantages in the early diagnosis, management, and repair of RAAAs, they still represent a challenging emergency, with mortality rates reaching up to 80% [9,19]. Surely, patients’ centralization and the widespread use of EVAR have improved overall survival [18,20]. As far as RAAA is concerned, unmodifiable factors such as female sex and elderly age have been well investigated and are known to negatively influence perioperative mortality [21,22]. However, a recent metanalysis reported that the 30-day and one-year mortality rates for RAAA repair in octogenarians are similar to the outcomes at all ages, with a significant survival advantage of EVAR over OSR [23].

Patients should, therefore, not be denied treatment based on these parameters alone, but the decision has to be individualized, taking into account other patient features before deciding to proceed with surgery. On this basis, several preoperative risk stratification scores have been created to predict outcomes after surgery. The presence of a well-performed preoperative risk score represents a useful tool, not only to give physicians a gist about what the outcome might be and facilitate the communication with patients and relatives, but mostly to guide the decision-making process.

The period between 1980 and 1990 witnessed the first attempt to create a preoperative risk model to predict early mortality in patients undergoing OSR for RAAA with the Glasgow aneurysm score (GAS). The risk score was calculated taking into account only the five variables of age, shock, and myocardial, cerebrovascular, and renal disease. Subsequent evaluation of the scoring system showed that the mortality rate increased in proportion to the score. The inclusion of only a few variables, which were readily accessible for patients arriving at an emergency unit in shock, seemed to render it a simple method for risk stratification [24].

In 1996, Hardman et al. [25] proposed the homonymous index, having identified five parameters: age, creatinine level, loss of consciousness after arrival, hemoglobin levels, and electrocardiographic signs of ischemia. One point was attributed to the presence of each variable and correlated with the mortality rate.

Two years after the GAS, the Vancouver Scoring System (VSS) was developed, including age, reduced level of consciousness, history of myocardial infarction, history of collapse, and preoperative cardiac arrest as predictors of death. These variables were entered into an equation where the probability of death was estimated using a mathematical formula [26]. Despite the subsequent validation of this score, its calculation seems to be too time-consuming, especially in emergency settings.

Subsequently, the group of Portsmouth elaborated the RAAA Physiological and Operative Severity Score for enUmeration of Mortality and morbidity (RAA-POSSUM). They modified the conventional POSSUM score that consisted of two components, a physiological score involving 12 preoperative physiological variables, and an operative score containing 6 operative variables, in order to adapt it to the specific case of RAAA. A regression equation was used to convert these raw scores into a predicted probability of death [27]. The limit of this model concerns the difficulty of calculation and the inclusion of intraoperative variables that may overestimate the number of patients at risk of death compared to the Hardman index [28].

In 2007, Tambyraja et al. [29] evaluated all the abovementioned scoring systems, concluding that none has been shown to have consistent or absolute validity, and that there were no individual or combination of variables that could accurately and consistently predict outcomes. For this reason, they described a new score named the Edinburgh Ruptured Aneurysm Score (ERAS), where the presence of three preoperative risk factors, easily assessable in emergency settings, corresponded to 80% of mortality. Compared to the Hardman Index, GAS, and RAAA-POSSUM, ERAS accurately stratified the risk, but needed further validation. However, all these scoring systems were developed exclusively for OSR and have largely fallen out of favor for this reason, as well as for the criticism that they fail to properly characterize mortality for the highest-risk patients [30].

Aiming to identify patients at higher risk after OSR, the Vascular Study Group of New England (VSGNE) developed and validated a practical risk score for in-hospital mortality with only four variables. Patient stratification according to the VSGNE risk score accurately predicted mortality and identified those at low and high risk for death [31]. Unlike the previous indexes, in the VSGNE score, the intraoperative variable of suprarenal clamping plays a central role in risk estimation of death, testifying both to the importance of intraoperative technical aspects in determining outcomes and the limits of their relative usefulness for preoperative decision-making.

The new era of mortality risk scores started with the Dutch Aneurysm Score (DAS), followed by the Harborview risk score (HRS) (Table 2). All previous risk scores have the limit of either including intraoperative variables in their formulas or not taking into account the increasingly widespread use of EVAR for RAAA. Indeed, the DAS and HRS have been developed to overcome these limitations. The first one was developed and externally validated, allowing surgeons to estimate the risk of death with four variables available prior to surgery. The DAS showed superior discriminative performance compared with the GAS in the Dutch population. This prediction model identified either low-risk patients in whom an intervention was likely to be beneficial, and patients at high risk of dying, in whom withholding an intervention might be considered [32]. The second one identified as the most predictive factors of mortality age, creatinine concentration, pH, and systolic blood pressure, allowing the accurate prediction of 30-day mortality. It also seems to have an impact on the clinical decision-making process by adding prognostic information to the decision-making process of transferring patients to a tertiary care center and to aid in preoperative discussions with patients and relatives [33]. A recent study by Hemingway et al. [34] confirmed the validity of the HRS in predicting 30-day mortality in a prospective consecutive series of modern patients with RAAAs.

Despite their accuracy in predicting mortality in patients undergoing EVAR or OSR for RAAA, neither DAS nor HRS are 100% reliable. Indeed, a comparison between widely used risk scores demonstrated that the performances of the tested models for the prediction of mortality were comparable, and an almost perfect prediction is needed to withhold intervention, but no existing scoring system is capable of that [35]. For this reason, all risk scores should never be used alone, without consideration of other potential factors that could influence patients’ short- and long-term prognosis. Their usefulness resides more in the important information they can provide, which can help clinical decision-making before treatment, rather than in giving a precise gauge of perioperative mortality in order to deny surgical treatment in selected patients. Briefly, given the highly morbid and resource-intensive nature of RAAA repair, risk stratification tools are important adjuncts that can guide physicians, patients, and families through challenging decisions.

Once a rapid assessment of a patient’s clinical condition has been performed, vascular surgeons have to face the main decision about the best treatment, choosing between OSR and EVAR. Although a significant number of patients die before reaching the hospital, OSR has been the gold standard for RAAA treatment over the years, with non-negligible mortality rates [36,37]. In contrast, EVAR has largely become the first-line treatment for intact abdominal aneurysms, but its role in an emergency setting has been extensively debated [38].

The first successful attempt to endovascularly repair an RAAA was reported in 1994 [39]. By that period, a lot of studies concentrated on comparing EVAR vs. OSR, with conflicting results, especially as to what concerns the real benefits of EVAR in the perioperative period and its durability [40,41]. Surely, an endovascular strategy implies the need for preoperative high-quality imaging for planning and knowledge of specific anatomical features to safely place the graft. Furthermore, Kontopodis et al. [42] demonstrated that patients with hostile anatomy treated by EVAR had a significantly higher death rate in follow-up than patients with a friendly aortic anatomy, whereas for OSR, the survival was similar in patients with hostile anatomy and those with friendly anatomy. Despite these possible limitations for emergent settings, in the wake of satisfactory results obtained with elective EVAR and the development of newer devices and techniques in this field, the proportion of RAAA repairs performed by endovascular technique increased. Furthermore, EVAR implies lower hemodynamic shifts that could potentially benefit survival after RAAA, especially in those patients already hemodynamically unstable. Another reason to explain this reducing trend in mortality due to EVAR could be a relatively better selection of patients.

Along with this ongoing debate, some data supporting EVAR strategy have been extracted from the Medicare inpatient dataset. The study showed an increased survival after RAAA of patients who were treated with EVAR as compared to those treated with OSR during the 4 years following after intervention [43]. The further significant improvement in the survival of RAAA patients treated endovascularly was due to a better distribution and variety of endovascular devices available, as well as increasing surgeon experience. The learning curve for elective EVAR is the cornerstone to achieve better outcomes after emergent EVAR. Indeed, survival rates of endovascularly-treated RAAA increase proportionally with the surgeon’s volume in both elective and ruptured AAA procedures. The same concept of surgeon experience could be also referred to for open repair. However, there are other factors that contribute to the successful management of RAAA that are outside the surgeon’s competence. Early diagnosis, an efficient transfer system between hospitals, the prompt activation of the whole vascular team, and an anesthesiologic team skilled in peri- and postoperative management of RAAAs are all variables that undoubtedly contribute to improved outcomes [44].

In order to achieve stronger evidence regarding the best choice between EVAR and OSR, in 2014, the IMPROVE (Immediate Management of Patients with Ruptured Aneurysm: Open Versus Endovascular Repair) randomized controlled trial (RCT) was developed. Due to its nature as an RCT, it had a real-world design, and hence was fundamentally different from the previous studies. The IMPROVE trial showed an overall 30-day mortality of 35.4% in the endovascular strategy group and of 37.4% in the open repair group. After adjustment for several variables, no difference in 30-day mortality existed between the two techniques [45]. Therefore, the primary result of this RCT showed no consistent survival advantages attributed to endovascular repair, and the question remains whether these findings are representative enough of a real-world population. Surely, the IMPROVE trial needed to be corroborated by further investigations. Contrarily, a recent population-based study showed that a routine practice of OSR appears to provide better results than EVAR with respect to survival after RAAA [46]. Specifically, patients treated in hospitals that perform only open repairs of RAAAs had improved short-term survival after controlling for important risk factors, such as hemodynamic instability and loss of consciousness. It is understandable that surgeons exclusively performing open repair can achieve better results than those who do these procedures only sporadically when patients are not suitable for EVAR. However, this study did not support the routine use of one practice over another.

Rather than the superiority of one treatment over another, all studies present in the literature seem to highlight a potential drawback to an exclusive endovascular-first approach. Vascular surgeons and hospitals with decreasing exposure to open RAAA cases are progressively losing the skills and experience needed to achieve satisfactory clinical outcomes. Although EVAR has become the most used technique of the endovascular-first era and has relatively better results in the short- and mid-term periods, its long-term efficacy and durability has yet to be determined. Furthermore, there is still a not negligible percentage of patients with RAAA unsuitable for EVAR. For these reasons, we cannot forget that OSR often represents the best choice, especially in cases where anatomical and technical difficulties may increase the risk of unsuccessful endovascular procedures, which by definition should be minimally invasive.

To conclude, the choice between EVAR and OSR has to be customized for each individual patient, taking into account the whole preoperative setting and assessment. Only centers with great experience in both open and endovascular surgery should treat RAAA, reinforcing once again the importance of centralization in order to achieve the best outcomes for such demanding conditions.

Besides an accurate preoperative assessment and a choice of treatment tailored to the individual patient, there are undoubtedly other factors that could affect survival after intervention. As already mentioned, risk scores are not completely reliable, because they do not take into account possible intraoperative variables such as the surgeon’s skills and the patient’s ability to recover. Therefore, it is essential to know and consider intraoperative factors that could influence the success of the intervention.

**Figure 3:**
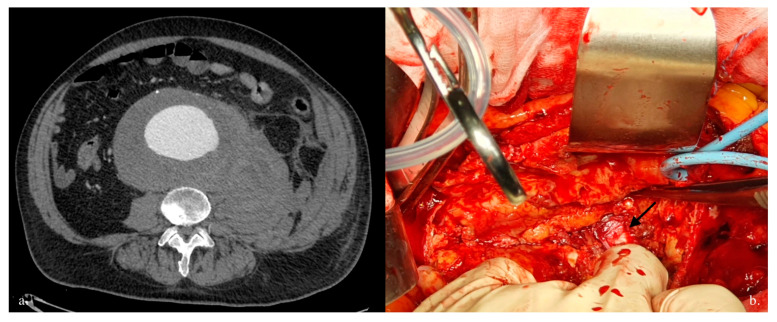
(**a**) CTA shows a postero-lateral AAA rupture with retroperitoneal hematoma. (**b**) The picture shows an open surgical repair of an RAAA with vertebral exposure (black arrow). CTA, Computed Tomography Angiography; AAA, abdominal aortic aneurysm; RAAA, ruptured abdominal aortic aneurysm.

The first attempts to investigate possible variables occurring during treatment were made in the 1990s, and were about the type of rupture, intraoperative hypotension, and whether total blood loss influenced survival the most [47]. These three factors are obviously interconnected, because an anterior aortic rupture leads to a massive blood loss more quickly with hemoperitoneum, and consequently to significative hypotension. A more recent analysis showed an operative and in-hospital mortality for RAAA patients of 22.9%, compared with 1.9% for that of non-ruptured AAA patients. The mean hemoglobin level was significantly lower in the death group than in the survival group, and intraoperative bleeding volume was higher for dead patients [48]. These findings underline the vital importance of intraoperative bleeding management as one of the main predictors of perioperative death (Figure 3). In this context, the surgeon plays a central role, reducing the time to achieve clamping or exploiting the resuscitative endovascular balloon occlusion of the aorta (REBOA) as a bridge to definitively control hemorrhage [49].

Only subsequently did Markovic et al. [50], aiming to define relevant intraoperative prognostic factors influencing outcomes in patients undergoing OSR for RAAA, highlight that the duration of clamping along with operation time and type of reconstruction were negative predictors of perioperative mortality after surgery. Obviously, the duration of surgery is directly linked to a prolonged aortic cross-clamping and to the need for more complex arterial reconstruction. In this analysis, no mention has been made about the type of clamping, which remains one of the most debatable issues in OSR for RAAA. Surely, the location of aortic cross-clamping is determined by the extension of aneurysmal degeneration and implies very different risks at baseline even in elective settings. Therefore, in the presence of infrarenal RAAA, with enough space at the level of the proximal neck, an infrarenal clamping is recommended. In fact, by guaranteeing a normal blood flow in renal arteries during the entire operation, it reduces the impact on kidney function, the occurrence of acute kidney injury (AKI), and the need for renal replacement therapy in the perioperative time, unlike what is observed with suprarenal clamping [51,52]. Conversely, for patients with ruptured juxtarenal aneurysms, supraceliac clamping enables safe and easy anastomosis to the healthy aorta, preventing late anastomotic aneurysm formation, which frequently occurs after inadvertent anastomosis of the graft to a diseased portion of the aorta. However, it cannot be ignored that major ischemia-reperfusion aggression, along with a significantly greater left ventricular afterload increase, are associated with supraceliac clamping. Therefore, whenever the type of rupture allows it, higher levels of aortic cross-clamping should be avoided, in order to reduce the reperfusion’s damage and achieve better outcomes.

First of all, due to its lower invasiveness, the EVAR technique eliminates the complications that can occur during laparotomy, minimizes hypothermia, and can be performed with the patient under local anesthesia. Secondly, EVAR should reduce intraoperative blood loss that inevitably would lead to hypotension and multiorgan hypoperfusion, with an increasing risk of intestinal ischemia. In fact, OSR seems to be associated with a threefold increase in the odds of bowel ischemia (BI) compared with EVAR. In addition, procedures performed during OSR, such as a transperitoneal approach, supraceliac clamping, a reimplanted inferior mesenteric artery, a long operative time, and the requirement for >1 U of blood transfusion are predictors of BI [53]. Furthermore, the use of REBOA during EVAR reduces the time to achieve the clamping, and the aortic stent graft can be deployed while the aorta is continuously clamped from a transfemoral approach even in cases of circulatory collapse [54].

However, EVAR is not completely free from complications, and cannot be performed in any case. Indeed, the development of abdominal compartment syndrome (ACS), which is one of the most frightening complications after the repair of RAAAs, seems to be associated with increased mortality, especially in EVAR-treated patients. The higher intraoperative blood product requirements associated with ACS in EVAR suggests that one potential cause of early ACS is continued hemorrhage from the lumbar and inferior mesenteric arteries through the ruptured aneurysmal sac. For this reason, open ligation of these vessels should be considered in patients developing early ACS after EVAR for RAAA [55]. Furthermore, not all patients are suitable for EVAR because of challenging anatomies. Indeed, performing EVAR while not following its instructions for use (IFU) yielded an inferior in-hospital survival rate compared to on-IFU EVARs. When compared with matched patients undergoing OSR with infrarenal or suprarenal clamping, survival was no different from off-IFU EVAR [56]. For sure, the comparison between mininvasive and open techniques in any field of surgery is difficult to make, mostly concerning the fact that the selection of patients eligible for each of the two techniques is itself a statistical bias that can hardly be overcome. Specifically, patients with more hostile anatomies, such as short, angulated, calcified, or tapered aortic necks are not suitable for EVAR, but are only eligible for OSR. The fact that OSR is mostly delivered to patients who are otherwise untreatable constitutes itself a factor that contributes to the poorer outcomes of open surgery.

Awareness of the discussed intraoperative factors affecting early mortality for both OSR and EVAR may allow a more targeted surgical strategy that may well lead to improved survival in RAAA patients. Furthermore, therapeutic efforts by the whole aortic team should concentrate on intraoperative variables that are possible to correct, leading to better survival for RAAA patients.

The potential superiority of rEVAR in reducing mortality over rOSR has been largely investigated among the scientific community [57,58,59,60,61].

The results of the analyzed studies are summarized in Table 3.

In any case, an EVAR-first approach is not suitable for all patients presenting with RAAA. Indeed, EVAR outcomes depend on several factors and, for sure, a lack of experience or skills could be greatly exacerbated when attempting to urgently treat an unstable patient [62,63]. For this reason, both OSR and EVAR should be performed in high-volume centers, in order to offer the best treatment and keep up skills in both approaches. Indeed, high-volume centers have better outcomes, regardless the approach used, and that is the reason why centralization is like a “first-line treatment”.

EVAR usually has lower rates of short-term complications, but more mid-term reinterventions, mainly related to endoleaks. When OSR is performed, pulmonary complications (pneumonia, respiratory insufficiency) are the most common ones (42%), followed by cardiac complications (18%), acute kidney injury (17%), ischemic colitis (9%), and wound complications (7%). Postoperative acute respiratory failure (ARF), MI, and MOF are reported to be predictors of in-hospital mortality, although the main common complication is hospital-acquired pneumonia (HAP) [64]. For this reason, the optimization of respiratory exchanges is mandatory.

After an intervention, the coagulation status could be affected, so that a prompt rebalance is compulsory. A complete neutralization with protamine sulfate of intraoperative heparinization could be a first-line treatment. Adequate fluid administration and renal function evaluation should be maintained during the immediate postoperative period. In addition, an evaluation of lower limb status is mandatory, especially when an OSR is performed.

Bowel ischemia is one of the most threatening complications due to its high mortality rates. Whenever emergency colonic resection is required, mortality rises up to 50% [65]. It is unclear whether there is a difference in bowel ischemia or colectomy rates between EVAR and OSR. However, patients potentially develop this condition within 7 days from both procedures.

Abdominal compartment syndrome is defined as a “condition in which the increased pressure in an inextensible compartment leads to decreased blood flow to abdominal organs determining ischemia and dysfunction and may evolve into permanent loss of function”.

Abdominal compartment syndrome (ACS) lacks definition consensus. ESVS guidelines describe it as a “sustained intraabdominal pressure (IAP) > 20 mmHg (with or without an abdominal perfusion pressure < 60 mmHg), that is associated with new organ dysfunction/failure”. In the literature, it has been described to occur in 6–55% of patients with RAAA. Monitoring, in each emergent patient, the intrabdominal pressure is crucial; this is feasible through the most frequent urinary bladder pressure measurement, or gastric pressure or vena cava catheterization [66]. There is still no consensus on the adequate criteria and timing for decompression. Surely, intrabdominal pressure should not exceed 20 mmHg and abdominal decompression should be considered in the presence of increasing pressure, organ failure, or persistent abdominal hypertension [67].

Over the past years, the concept of temporary abdominal closure with impermeable mesh or Silastic sheeting of a vacuum-assisted closure has been developed. Patients who needed mesh closure had a higher mortality rate than patients who underwent primary closure. However, patients who underwent mesh closure during the initial operation had lower mortality rates and were less likely to develop MOF than patients who underwent mesh closure after a second operation in the postoperative period for abdominal compartment syndrome [68]. In a retrospective analysis by Kimball et al. [69], preoperative hypotension, blood loss of at least 6 L, or intraoperative resuscitation with at least 12 L negatively predicted mortality. They recorded a statistically significant survival benefit in the first 24 h after surgery for patients treated with a vacuum-pack technique. Delayed primary fascial closure should be performed as soon as possible to reduce risk of complications such as incisional hernias and infections. According to recent data, the vacuum-assisted wound closure with or without mesh interposition could achieve good results in terms of decompression, reducing morbidity, mortality, and local infective complications [70].

In conclusion, increased abdominal pressure is a negative predictor of survival after OSR for RAAA. Measurement of intrabdominal pressure is recommended after intervention and, in case of high levels in combination with organ dysfunction, decompressive surgery should immediately be performed. Temporary abdominal closure systems can positively influence outcomes.

Endoleaks are the most common complications associated with EVAR, with an incidence of up to 30% [71]. In the late postoperative period, there is a global consensus about the treatment of type I or III endoleaks when feasible. Type Ia endoleaks can be treated through aortic cuff extension, especially if due to previous misdeployment or undersized grafts. Another option is balloon angioplasty via a large-caliber balloon to achieve optimal graft sealing. In case of technical failure, endovascular repair with bare metal stents can be applied to secure the sealing zone. Type Ib endoleaks can be solved through iliac extender limbs, bare metal or covered stents, and embolization of internal iliac arteries. Type III endoleaks develop whenever the endograft fails to maintain structural integrity. This happens because of the dehiscence of modular graft components (type IIIa) or as a result of tears in the endograft structure (type IIIb). Most type III endoleaks can be managed with an endovascular approach by deploying a modular endograft followed by angioplasty to achieve optimal sealing.

Once a patient has survived for the first 30 days after intervention, survival rates become similar to those who underwent elective OSR [72]. For survivors, age, but not comorbidities, has a significant influence on long-term survival. Regarding octogenarians and nonagenarians, perioperative mortality is as high as 50% after OSR, with no significant differences between sexes and worse survival with increasing age. However, after 90 days from intervention, long-term survival in the oldest cohort is surprisingly good, reaching up to 50% after 5 years, similar to the general population. All these findings suggest that OSR could be performed with good short- and long-term results, especially in young and hemodynamically stable patients.

Several studies reported lower 30-day mortality rates for rEVAR compared to rOSR, but with higher reintervention rates and long-term mortality. Whenever EVAR is performed, the long-term results depend tightly on the availability of a suitable endoprosthesis. Indeed, performing an off-IFU EVAR leads to higher complication and reintervention rates during the same amount of time.

Nowadays, emergency EVAR represents the first-line treatment for RAAA in centers where endoprosthesis and hybrid theatres are available. This is due to its significant perioperative benefits compared to OSR, especially in patients with hemodynamic instability and higher preoperative risk scores. However, EVAR continues to show high reintervention rates, mainly related to graft complications, such as endoleaks, kinking, sac enlargement, and endograft infections [73]. This implies that postoperative graft surveillance and infection monitoring are critical after EVAR.

Proximal and distal relining are valid techniques to treat type Ia and Ib endoleaks. Collateral aortic branches and aneurysmal sac embolization are frequently performed for type II endoleaks. However, secondary endovascular procedures may fail, so that surgical conversion becomes mandatory to achieve effective and durable outcomes. The management of such complex cases can be challenging and technically demanding, with high mortality rates [74].

Emergency EVAR is a valid approach in RAAA patients with suitable anatomy, especially when performed inside the IFU and in experienced centers. Nevertheless, the higher risk of complications compared to EVAR in an elective setting warrants long-term follow up.

## 5. Conclusions

RAAA remains a life-threatening condition with high mortality rates in the acute phase. The high risk of secondary procedures in the postoperative period and aneurysm-related re-interventions are mainly observed following EVAR. Treatment optimization may lead to a more targeted approach in terms of THE choice between OSR and EVAR. Effective screening programs, surveillance, and risk factor control may play a role in reducing RAAA development, but further research is necessary to better understand the interaction of the different aspects involved in the development of the disease.

## Figures and Tables

**Table 1:** Summarizes PICOS criteria on which the review was performed.

PICOS Criteria to Develop the Research	
Participants	All patients with ruptured abdominal aortic aneurysms (rAAA) treated in emergency settings, with treatment performed in high-volume centers.
Intervention	All emergency open surgical repair (rOSR) and endovascular abdominal aortic aneurysm repair (rEVAR) performed in the analyzed period. All preoperative score models used to predict 30-day mortality and to guide decision-making process.
Comparison	All studies investigating the comparison between rOSR and rEVAR (e.g., IMPROVE trial). Main intraoperative factors influencing survival in patients undergoing either rOSR or rEVAR.
Outcomes	All studies discussing optimal treatment strategies, complications, short-term and long-term outcomes after both rOSR and rEVAR.
Study design	All prospective and retrospective studies (clinical cases and case series have been excluded).

**Table 2:** The table summarizes the mentioned risk scores and their interpretations.

Model	Formula	Interpretation
GAS (Glasgow Aneurysm Score)	Age + 17 for shock + 7 for myocardial disease + 10 for cerebrovascular disease + 14 for renal disease	Score > 95 = mortality risk > 80%
Hardman Index	Age > 76 years Hemoglobin < 9.0 g/dL Creatinine > 190 mmol/L, Electrocardiographic ischemia Loss of consciousness Score from 1 to 5 depending on number of five risk factors present	Score 0 = 16% mortality Score 1 = 37% mortality Score 2 = 72% mortality Score ≥ 3 = 100% mortality
VSS (Vancouver Scoring System)	E^x^/(1 + E^x^), where x = (−3.44) + [sum of coefficients of significant variables] Variable Coefficient Age 0.062 × age Reduced consciousness Yes: 1.14 Reduced consciousness No: −1.14 Cardiac arrest Yes: 0.6 Cardiac arrest No: −0.6	Result of formula is the calculated mortality risk
RAA-POSSUM (RAAA Physiological and Operative Severity Score for enUmeration of Mortality and morbidity)	In(R/1 − R) = −4.9795 + (0.0913 × physiological score) + (0.0958 × operative severity score) R = risk of death	Result of formula is the mean predicted risk of death
ERAS (Edinburgh Ruptured Aneurysm Score)	Preoperative Glasgow Coma Scale score < 15 Preoperative systolic blood pressure < 90 mm Hg Hemoglobin level < 9 g/dL Score from 1 to 3 depending on number of three risk factors	Score ≤ 1 = 30% mortality Score 2 = 50% mortality Score 3 = 80% mortality
VSGNE (Vascular Study Group of New England)	Age > 76 years: 2 points Cardiac arrest: 2 points Loss of consciousness: 1 point Suprarenal aortic clamping: 1 point	Score 0 = 8% mortality Score 1 = 25% mortality Score 2 = 37% mortality Score 3 = 60% mortality Score 4 = 80% mortality Score ≥ 5 = 87% mortality
DAS (Dutch Aneurysm Score)	(age × 0.74) + (systolic blood pressure [mm Hg]/10 × −0.12) + (1 for cardiopulmonary resuscitation) + (hemoglobin [g/dL]/10)^3 × − 1.27^) ln (odds): −4.73 + DAS 30-day death rate = exp(ln(odds))/(1 + exp(ln(odds)))	Result of formula is mortality risk
HRS (Harborview Risk Score)	Age > 76 years pH 2 mg/dL Creatinine > 2 mg/dL Any episode of hypotension, defined as systolic blood pressure < 70 mmHg 1 point for each of four preoperative variables when present	Score 0 = 14.6% mortality Score 1 = 35.7% mortality Score 2 = 68.4% mortality Score ≥ 3 = 100% mortality

**Table 3:** Summary of the short-term results of both rEVAR and rOSR.

Study	Cohort	30-Day Mortality OSR (%)	30-Day Mortality EVAR (%)	*p*-Value
Veith et al. [59], *Ann. Surg.* 2009	1037	36.3	21.2	<0.0001
Nedeau et al. [61], *J. Vasc. Surg*. 2012	74	49	15.7	0.008
Kapma et al. [60], *Br. J. Surg*. 2014	116	25	21	0.002
Powell et al. [45], *BMJ* 2014	613	37.4	35.4	0.620
Gupta et al. [58], *J. Vasc. Surg*. 2014	1447	52.8	35.6	<0.0001
Jones et al. [57], *J. Vasc. Surg*. 2022	376	29.9	27.7	0.687

EVAR, EndoVascular Aortic Repair; OSR, Open Surgical Repair.

## Data Availability

Not applicable.
